# Social determinants of health and long‐term conditions in people of Black African and Black Caribbean ethnicity living with HIV in London: A qualitative study

**DOI:** 10.1111/hex.14055

**Published:** 2024-04-26

**Authors:** Vlad Kolodin, Birgit Barbini, Denis Onyango, Rachel Musomba, Jia Liu, Rachel K. Y. Hung, Elena Nikiphorou, Lucy Campbell, Frank A. Post, Shema Tariq, Heidi Lempp

**Affiliations:** ^1^ Department of Sexual Health and HIV King's College London London UK; ^2^ Department of Sexual Health and HIV King's College Hospital NHS Foundation Trust London UK; ^3^ African Advocacy Foundation London UK; ^4^ GKT Hospital, School of Medical Education, Centre for Education, Faculty of Life Sciences and Medicine King's College London London UK; ^5^ Department of Inflammation Biology, Centre for Rheumatic Diseases, Faculty of Life Sciences and Medicine, School of Immunology and Microbial Sciences King's College London London UK; ^6^ Centre for Clinical Research in Infection and Sexual Health, Institute for Global Health University College London London UK

**Keywords:** Black African, HIV infection, long‐term conditions, qualitative research, social determinants of health

## Abstract

**Background:**

People living with human immunodeficiency virus (HIV) are disproportionately impacted by socioeconomic deprivation and are at increased risk of developing other long‐term conditions (LTCs). These illnesses require transformative action to tackle the adverse effects on their health. Data on lived experiences of LTCs among people living with HIV of Black African and Black Caribbean ethnicities are sparse, and how people with LTCs are impacted by social determinants of health (SDoH).

**Methods:**

Through a phenomenological study design this qualitative study, conducted in 2022, comprised four focus group discussions (FGDs) with 20 people of Black ethnicities living with HIV were purposively invited from a community organisation (CO) in London, including four semistructured interviews with CO staff. Following transcription, qualitative data were analysed thematically and measures to validate the findings were implemented.

**Results:**

The findings are presented in terms of the following four levels of SDoH: (1) individual determinants (such as the impact of SDoH on lifestyle modification and self‐management); (2) interpersonal determinants (such as positive experiences of accessing healthcare for LTCs); (3) clinical determinants (such as care pathway barriers) and (4) systemic determinants (such as systemic barriers related to race/ethnicity).

**Conclusions:**

It is necessary to provide ongoing and interactive education to community members who live with HIV, focusing on risks and management of LTCs. Additionally, individuals would benefit from support to navigate increasingly complex and fragmented health services. Health Service staff require cultural competence when caring for patients of Black African and Black Caribbean ethnicities with complex health and psychosocial needs.

**Patient or Public Contribution:**

The research team collaborated with an HIV CO in South London from the very start of the project to agree the study design and learn about the realities of their daily lived experiences. Community collaborators helped to develop the semistructured interview and FGD topic guides, and were directly involved in the data gathering, analysis and validation.

## 1 INTRODUCTION

The widespread availability of effective antiretroviral therapy (ART) has transformed human immunodeficiency virus (HIV) from a condition which was almost universally fatal, to a long‐term condition (LTC) with almost normal life expectancy.^1^ However, this shift towards HIV as an LTC has meant that people living with HIV increasingly experience age‐related conditions and multimorbidity. Multimorbidity (defined as the presence of two or more LTCs) has been identified as a key challenge for people with HIV, along with lower quality of life, and HIV‐related stigma.^2^

The importance of social determinants of health (SDoH) is that addressing these determinants is of fundamental importance for improving health and reducing longstanding inequities in health, which requires action by all sectors and civil society.^3^ The World Health Organisation (WHO) has defined the SDoH as the nonmedical factors that influence health outcomes. According to the WHO, SDoHs are ‘the conditions in which people are born, grow, work, live, and age, and the wider set of forces and systems shaping the conditions of daily life. These forces and systems include economic policies and systems, development agendas, social norms, social policies and political systems. The SDoH have an important influence on health inequities—the unfair and avoidable differences in health status seen within and between countries’ (https://www.who.int/health-topics/social-determinants-of-health#tab=tab_1).

SDoH can, for example, include the following types of determinants: income and social protection; education; unemployment and job insecurity, working life conditions; food insecurity; housing, basic amenities and the environment; early childhood development; social inclusion and nondiscrimination; structural conflict and access to affordable health services of decent quality. It follows that SDoH can be identified at the individual, interpersonal, clinical and structural levels.

They have a significant impact on an individual's quality of life and access to healthcare. Race and ethnicity are also strongly associated with health and wellbeing, mediated predominantly by SDoH, including structural racism. For example, both Covid‐19 pandemic and LTCs, such as diabetes, chronic kidney disease and mental illness, disproportionately affect racially minoritised people.^4^, ^5^, ^6^

HIV is associated with adverse SDoH. In 2022, nearly 1000,000 people accessed HIV care in the United Kingdom,^7^ of whom 30% were of Black African, and 3% of Black Caribbean ethnicity. A recent meta‐analysis of studies investigating material deprivation and HIV outcomes in high‐income settings found an association between various SDoH and retention in HIV care, adherence to ART, and viral suppression.^8^ Furthermore, racially minoritised people living with HIV are more likely than those of White ethnicity to be diagnosed late, to disengage from care, and to experience viral rebound.^4^

Multimorbidity has been shown to be associated with income, race and education amongst people living with HIV.^8^, ^9^ This is of particular importance given the high levels of socioeconomic deprivation amongst people living with HIV. In the United Kingdom, it is estimated that one‐third of individuals living with HIV lack the resources to consistently meet their basic needs, with a notable overrepresentation of people from racially minoritised communities.

Literature about the relationship between LTCs and SDoH in HIV in high‐income settings is sparse. Furthermore, few qualitative studies have explored lived experiences of LTCs amongst people living with HIV, and how they are shaped by SDoH. Such data have the potential to elucidate the complex interplay by which SDoH lead to poor health outcomes, as well as providing insight into the challenges and solutions within communities. This qualitative study was nested within a larger quantitative study investigating SDoH and co‐ and multimorbidity in people of Black ethnicities living with HIV in London.^9^

This study seeks to address this research gap by exploring qualitatively the lived experiences of LTCs amongst people living with HIV of Black African and Black Caribbean ethnicities, and how they are impacted by SDoH. Specific objectives were to explore, amongst people of Black ethnicities living with HIV: (i) awareness of LTCs; (ii) impact of SDoH on the development and self‐management of LTCs; (iii) health care access for LTCs.

## 2 METHODS

This was a qualitative study comprising focus group discussions (FGDs) (*n* = 4) with people living with HIV of Black ethnicities in London recruited through our collaborating community organisation (CO), and semistructured stakeholder interviews (SSIs) with CO staff (*n* = 4). All FGDs and SSIs were conducted between May and July 2022, with sample size determined by data saturation.^10^

### 2.1 FGDs

Members of the collaborating CO were approached in person or by phone by co‐author (D. O.) and invited to participate in FGDs. Individuals were eligible if they were living with HIV, of self‐reported Black ethnicity, and aged 35–65 years. People were eligible regardless of gender and/or whether they were living with LTCs apart from HIV. FGDs were conducted either online (*n* = 1) using Microsoft Teams, or in person on the CO premises (*n* = 3) and co‐led by V. K. and a CO staff member (R. M.), a Black African woman with well‐established relationships with members. FGDs were audio‐recorded with consent, and contemporaneous notes taken by facilitators, lasting between 90 and 120 min. They were mixed gender groups, with same‐gender breakout discussions covering potentially sensitive topics such as reproductive and urological health, and menopause. All participants received £20 in recognition of their time and expertise.

The FGD topic guide was informed by existing literature^11^, ^12^, ^13^ and the experiential knowledge of the research team, in close collaboration with CO members. The FGD topic guide was further refined through discussion with CO staff and members. The FGD covered experiences of LTCs, the potential impact of SDoH on LTCs, knowledge and perceptions of LTCs, care seeking, attitudes towards prevention interventions, treatment, and self‐management (see Appendix A). Participants also provided sociodemographic information.

### 2.2 Semistructured interviews

FGDs were supplemented with SSIs with staff and trustees at the collaborating CO. Potential SSI participants were identified by the study team and CO and contacted by DO. SSIs were all conducted online by V. K., a white male researcher who had no previous contact with participants. SSIs lasted between 40 and 60 min and covered perspectives on the impact of LTCs on their service users, and potential strategies to address LTCs in this population. (see Appendix A) Interviewees provided sociodemographic information.

### 2.3 Data analysis

FGD and SSI audio recordings were transcribed verbatim by a professional transcription agency, and content double checked for accuracy. A phenomenological approach for the qualitative data analysis was undertaken,^14^, ^15^ a method that is fundamentally concerned with people's perceptions and subjective experiences of their reality, in a particular context and at a particular time.^14^

Two authors (V. K. and B. B.) carried out a reflexive, inductive thematic analysis^16^ using NVivo 12,^17^ each coding all transcripts. Reflexive thematic analysis is a flexible and iterative approach to qualitative data that allows researchers to explore and understand underlying themes, with specific attention to researcher subjectivity. A third qualitative external researcher (J. L.) analysed two randomly selected interviews and one focus group, leading to further refinement of themes.

FGD and SSI data were triangulated to assess convergence and deviation of findings between CO members and stakeholders.^18^ Data were presented to CO members and staff for respondent validation.^19^

### 2.4 Patient and public involvement

The study team collaborated with a partner CO which was set up in 1996 and is based in South London, an ethnically diverse area where 25% of the local population are of Black African or Black Caribbean ethnicities. The community‐led charity focuses on supporting and empowering diaspora communities to achieve better health and wellbeing. It has a comprehensive programme of services aimed at preventing HIV and supporting people living with HIV in Black communities in London. Two members of the CO were part of the study team, contributing to study design, data collection and analysis and its members and staff informed the development of the topic guides.

### 2.5 Reflexivity statement

The study team comprised qualitative and mixed‐methods researchers from multiple ethnic backgrounds (Black African, White European and East Asian). They were aged between 30 and 65 years old; three were cisgender female and two cisgender male. Most had academic and/or clinical experiences of working in the field of HIV in London.

## 3 RESULTS

Data from FGDs and SSIs are presented together, with pseudonymised accounts to illustrate identified themes. As an indication of how many participants contributed to each theme, ‘majority’ corresponds with more than 15, ‘most’ for more than half and ‘some’ indicates fewer than half.

Four FGDs were conducted with a total of 20 participants (*n* = 3–9) in each FGD (see Table 1 for participants' sociodemographic characteristics). Four stakeholders (one cisgender male and three cisgender females have worked at the CO between 4 and 10 years). Two were unable to take part due to other commitments.

**Table 1:** Sociodemographic characteristics of focus group attendees.

	Women (*N *= 11)	Men (*N *= 9)	Total
Age			
36‐45	4	1	5
46–55	3	2	5
56–65	4	6	10
Regions of birth			
Southern Africa	3	2	5
Eastern Africa	5	6	11
Central Africa	2	0	2
Western Africa	0	1	1
The Caribbean	1	0	1
Years living with HIV			
<10	1	0	1
11–20	3	1	4
>21	5	7	12
Not answered	2	1	3
Long‐term conditions (self‐reported)^a^			
HIV+ 1 long‐term condition	4	2	6
HIV+ >1 long‐term condition	5	3	8
Cardiovascular disease	6	3	9
Liver disease	1	0	1
Mental Illness	2	0	2
Diabetes	2	1	3
Neuropathy	1	1	2
NA^a^ (no long‐term conditions)	2	4	4
Years living with long‐term conditions^a^			
<5	1	0	1
6–20	3	1	4
>21	6	5	11
NA^a^	0	1	1
Highest level of completed education^a^			
No formal	0	1	1
Primary	1	0	1
Secondary	4	0	4
University	4	6	10
Employment^a^			
Fulltime	0	0	0
Unemployed	6	3	9
Part time	2	0	2
Voluntary	1	4	5
Shared HIV status with others^a^			
Family and/or friends only	1	0	1
GP, and family and/or friends	7	4	11
GP only	1	2	3
NA^a^ (not told anyone)		1	1

Abbreviations: GP, general practitioner; HIV, human immunodeficiency virus; NA, no LTC, apart from HIV; SDoH, social determinants of health.

a Missing data, participants did not complete all the sociodemographic information.

The qualitative data gathered during focus groups, complemented by the interviews is presented in relation to individual, interpersonal, clinical, and systemic determinant levels described above, and this framework is used to organise Table 2.

**Table 2:** Overview of the overarching themes and subthemes identified from the focus groups and interviews.

Overarching themes	Subthemes
Individual level determinants	Awareness of long‐term conditions Impact of SDoH on mental and physical health Impact of SDoH on lifestyle modification and contributing to self‐management
Interpersonal level determinants	Positive experiences of accessing healthcare for long‐term conditions
Clinical level determinants	Care pathway barriers Practitioner related barriers Covid pandemic‐related clinical barriers
Systemic level determinants	Systemic barriers related to race/ethnicity

Abbreviation: SDoH, social determinants of health.

### 3.1 Individual level determinants

#### 3.1.1 Awareness of LTCs

CO stakeholder participants highlighted the burden of LTCs amongst members living with HIV:

A gentleman, our service user … has got diabetes, high blood pressure, kidney disease, [high] cholesterol … and is going for a [kidney] transplant and for dialysis three times a week. [SSI participant 4]

However, a majority of FGD participants reported limited awareness of the risk of developing LTCs, especially in the context of HIV:

Some diseases, they just came like a surprise, because we never knew about them [LTCs] and get sick … I would say it's kind of like a lack of information, we were not aware about it. [FGD3 Female]

I think … the HIV might have a direct relationship with some of the conditions I have as the direct cause … I'm not too sure. Say for example I didn't mention the thinning of the bones, osteoporosis. My understanding is perhaps some of the antivirals are directly linked to that and … problems with sleepless … the neuropathy perhaps … the HIV diagnosis itself or maybe the medication would impact on somebody's mental wellbeing… [FGD4 Male]

CO stakeholder participants described how HIV is often the focus of attention for people living with HIV, and that proactive provision of information may improve awareness and prevention in these communities:

From the African context, once they [clinicians] are going to say [they are] HIV positive, your mind is consumed by that [the diagnosis]. You're no longer inquisitive of other [long‐term] conditions until it is too late, but if they [patients from African communities] had prior knowledge, perhaps they would have been equipped to know better how to look after themselves and prevent … experiences of the long‐term conditions. [SSI participant 2]

Furthermore, despite the relatively high prevalence of LTCs amongst people living with HIV, CO stakeholders reported that such conditions were rarely discussed within communities:

I've seen people along the years who came … to us with one illness and now after a couple of years we are seeing them getting other illnesses, and it's because maybe within the community, within the Black Minority Ethnic community there are things we don't talk about. [SSI participant 4]

#### 3.1.2 Impact of SDoH on mental and physical health

Many FGD participants highlighted the impact of socioeconomic factors on both their mental and physical health:

So really, I think while living with HIV, taking a lot of, taking medication for that, living with my own comorbidities of high blood pressure, and also being diagnosed with that you know what you are prone to diabetes, and also these aches and pains and, you know, looking after somebody [husband with HIV] … is really, you know, making me very, very, very, very stressed. [FGD1 Female]

And then because you don't have enough money to buy better food and drinks, which is low salts, then you are constantly consuming these high sugary drinks because they are cheap, because I don't have enough money to buy good ones, then diabetes comes along the line, I was pre‐diabetic myself and I found it was that [cheap drinks] [*laughs*]. [FGD2 Male]

I can categorically say there is a correlation between having no money and then gaining more weight, because when you have no money you go for the cheapest things that you can find … like there are some areas where they [residents] are so kind of influenced like, the way that people live there, they live longer and people in areas where there's deprivation, where there's poverty, and it can actually just be two bus stops away …. so yeah, definitely for me I can say that, and for us who come here [CO] I can relate to say whereby immigration can make you go mental. (FGD3 Female]

Okay, that time when I came here [London] that … I went to college, and I completed my law degree, the question is here, but after I … completed my law degree I went to the Bar, but even then, I don't get jobs, [so] I started drinking and start[ed] getting depressed. [FGD2 Male]

Many participants had migrated from African countries, with experiences of the UK immigration system, specifically delays in immigration decisions and resulting social and psychological uncertainty, shaping their health and well‐being:

You come here, you've got no‐one, you are starting a new life, you are trying to mingle around, you are trying to find your feet and all that; that can cause [poor] mental health, that can cause depression, [and] that can cause a lot of problems, and because what happens is, let's say, you have to wait for ten years before you get your paperwork [migration status], all this time your life is unbalanced, you don't know whether you're going, whether you're coming. [FGD3, Female]

CO stakeholder participants emphasised the contribution of housing insecurity, in the context of uncertain immigration status, to longer term physical and mental health, beyond HIV:

… first of all, … [if] a person is not stable mentally and does not have a place, a permanent place, you know, to live in [accommodation], then [this] does not only affect their [underlying HIV] condition but affects their whole life really, their whole health, their entire health is affected and that makes their long‐term health conditions worse. [SSI participant 3]

#### 3.1.3 Impact of SDoH on lifestyle modification and self‐management of LTCs

FGD participants identified diet and physical activity as key factors in the development and outcome of LTCs, however it was hard for some to implement these modifications:

It is cholesterol, which I have explored, and I feel that … the lack of [a healthy] diet, proper diet, eating properly and exercise, which I am not doing… [FGD2, Male]

Challenges experienced by people living with HIV and other LTCs become self‐perpetuating, further impacting their health negatively:

… it becomes a vicious circle … because of people's physical problems … they won't be able to [buy] fresh food or even bother and then so the type of food … they … take in [eat] … create this condition of obesity and it creates often high blood pressure … and all those conditions are also creating all of [the LTCs] they want to [avoid]. [SSI participant 2]

For some, there were financial barriers to exercise, for instance being able to attend a gym, and a perception that advice did not consider these barriers:

… what the doctors actually suggest will be so hard for us to start with, you know, even starting these exercises … it will be so difficult … we end up not even attempting all those advices … and I don't even know how to do it [exercise] slowly so that I could lose it [weight] slowly, you know. The gym is not free. And I'm not doing anything. [FGD 1 Female]

Furthermore, diet was often linked to longstanding cultural traditions, and therefore difficult to address:

… another [factor] that contribute[s] a lot [to developing LTCs] is the way we Africans cook. Most of us, we actually use a lot of oil in our food … yeah. So, this is another major factor that affect[s] our life … especially the cholesterol, high blood pressure and also this diabetes and obesity and everything. Even some cancers. [FGD1 Female]

These accounts highlight that addressing lifestyle factors to prevent or attenuate LTCs in these communities is not simply a matter of providing information and education. Many people were aware of the importance of diet and exercise, however social and cultural factors made it hard to adhere to medical advice. CO stakeholder spoke of the importance of healthcare providers understanding these barriers, and situating advice within these communities’ social and cultural contexts:

… from a clinical point of view, you tell them [members] ‘do this, do this, do this’, but from a practical point of view that's not translated [into their cultural context] … if they have grown up eating certain foods all their lives, how they then change to eat what you're telling them to eat is one thing. [Instead] how you adapt what they're eating to make it more healthy may be [better]. [SSI participant 1]

However, providing appropriate information about lifestyle, such as diet, can be an important lever to change:

… we are eating [food] from Africa, we are just eating what we see. But when you go for a training, the trainer will tell you ‘this is the amount of food, like five of fruit’. Until when I went to a training … that is why they [session lead] gave me [explained] the nature of the food I'm supposed to be eating. If I have not been going to that training, I wouldn't know that. [FGD4 Male]

### 3.2 Interpersonal level determinants

#### 3.2.1 Positive experiences of accessing healthcare for LTCs

Despite the numerous reported barriers to engaging with healthcare services for LTCs, a few participants described positive experiences of healthcare services, often as a result of support from their COs.

[In] 2013 precisely, I was diagnosed with a heart attack so, and it was treated in a Hospital where they gave me all the necessary support and ever since then [monitor] my blood pressure. [FGD4 Male]

CO stakeholders provided specific examples of the role COs can play in supporting people to manage HIV and LTCs.

What we've tried to do is introduce dieticians who are either from that [specific African] community or [are] really versed with the community and therefore you talk about the foods they eat and explain the calories or the implications of the different types of food and recommend substitutions or different ways still within the cultural context, as opposed to telling somebody to eat something they've never eaten. [SSI participant 1]

My experience of running a support group has meant that most Africans derive or get empowered through using this … this structure of sort of informal community settings. They will definitely benefit from that. [SSI participant 2]

### 3.3 Clinical level determinants

#### 3.3.1 Care pathway barriers

Many described navigating care pathways and onward referrals to specialist care for LTCs as becoming increasingly complicated; this was especially the case amongst women:

I'm the one who's suffering from it [pain], so if you talk about the care, yeah, it is there, but it's getting so complicated nowadays. It [NHS] was better off when I remember when we first started [came to this country]. If you complained to a GP, consultant, your GUM consultant, and say, ‘You know what, I'm having these problems’, they would straightaway refer you and say, ‘go for a scan’, immediately. They will refer you to a gynaecologist themselves, but now they'll tell you ‘that's not our job, you need to speak to your GP’. [FGD3 Female]

…. accessing them [medical consultations], like a General Practitioner has become hard [to obtain an appointment]. [FGD2 Male]

Navigating clinical care was further complicated by already living with a LTC, and difficulties in coordinating care amongst a range of healthcare providers:

… finding all of those health care providers become[s] a problem in itself and I think people simply give up and they will be resigned to living with those conditions. [SSI participant 2]

Once, referred to specialists, some described limited understanding of medical terminology, coupled with lack of confidence in asking questions to healthcare providers:

Even if you got it [understood the doctor's explanation], perhaps you've got a limited understanding, using jargon, you cannot sort of marry common man's language that [is] used with the medical language. [FGD4 Male]

For others, the ability to advocate for oneself was constrained by culturally bound views of doctors:

The problem we have as Africans, [is] we're not persistent. Whatever the doctor will tell you we find it very … we're a bit shy to like talk back to the doctor [and] say ‘no doctor, can you do this [test for example]?’ No, [we] just take whatever the doctor said. [FGD1 Female]

Whereas for some, lower educational status impacted confidence in navigating clinical consultation, ultimately affecting management of LTCs:

… for some people the level of education may be very low…they've never integrated fully into the [NHS] system here … advocating for themselves becomes an issue, so how they then manage these long‐term conditions is greatly affected. [SSI participant 1]

Care‐seeking for LTCs was further compromised by HIV‐related stigma.^20^ Most FGD participants reported self‐stigma following their HIV diagnosis, and the impact this had on their willingness to tell others in their community about their HIV status.

… I was hiding my medication because I have to receive people in my house, then when they walked away, I couldn't [find my HIV medication] … yeah, because it's not all the family who knows. Now even it [HIV medication] gives me another stress … you think people are looking [at you] … you are not the same [person]. [FGD1 Female]

The fear of stigma and discrimination as a result of their HIV status also affected their willingness to engage with health services:

It's [stigma] actually bigger than living with HIV … we [the CO] are trying … with this campaign [to] say ‘stigma is killing more people than HIV itself’ because it stops people from engaging [with NHS services] … it's a huge, it's a huge, huge issue. [SSI participant 1]

#### 3.3.2 Practitioner related barriers

Some FGD participants perceived a lack of attention from healthcare providers during routine non‐HIV consultations (i.e. for other LTCs):

… they [medical staff] give them [patients] … might be 5 minutes [or] 10 minutes, [but] they don't let you really explain everything [medical situation]. [FGD1 Female]

… [the] General Practitioner and [the] doctor [non‐HIV specialist in secondary care] … are not kind of looking at the person … when they [patients] are coming to say that ‘I have this problem’, they [medical staff] are not kind of really take it seriously to monitor what is going on… [FGD1, Female]

Some reported instances of HIV‐related stigma from NHS staff and discrimination within healthcare settings.

…NHS staff as well, so because I do outreach, I was at an outreach session, and I was talking some stuff about HIV. This nurse said, she goes, ‘people [who] have got it [HIV] through blood it's not their fault, but everyone else it's their own fault’, and I just looked at her and I was like, oh, this is NHS staff. [FGD3 Female]

We face challenges every day‐in day‐out. It doesn't stop … there was a woman in our support group … in hospital, the support staff doesn't even come [close] to her because [of her HIV diagnosis] … you can see that kind of stigma. [FGD4 Male]

Stigma within healthcare services had a particularly negative impact on people's readiness to engage with healthcare services, with potentially detrimental effects on the management of LTCs, which often require long‐term monitoring and treatment:

But mainly also stigma comes from professionals as well … the professionals not understanding the person's background or perception on things, so it's the way a professional can approach a person in asking what is affecting them can either put off the person or encourage them to come back. [SSI participant 3]

#### 3.3.3 COVID‐19 pandemic‐related clinical barriers

This study was conducted in Summer 2022, when NHS services had undergone significant disruption as a result of the COVID‐19 pandemic. Many HIV and non‐HIV related services had been reconfigured, with nonessential appointments deprioritised and most services provided remotely. In 2022, many NHS services remained affected by the pandemic.

Some FGD participants reported HIV clinic appointment consultations as problematic, not only because of disruptions to HIV care but also the impact on screening and monitoring of other LTCs (which are routinely undertaken within HIV clinics).

My smear test has been cancelled now; I think this is the fourth time, and I was due to be done in March. [FGD3 Female]

For many, the switch to remote appointments meant the loss of continuity with staff they had built relationships with over years and difficulties establishing rapport over the phone:

Even … with my bloods, like last year, with the HIV bloods for the past two years, every time they [HIV clinic staff] give an appointment, they cancelled it. They said they didn't have any bottles to take bloods, it was kind of frustrating. They cancelled, then you're getting a phone call with the doctor, which is not really great, because I used to see my doctors face‐to‐face before that. Even my HIV doctor. And [now] I've got a new doctor, and I've never met her yet, just on the phone. [FGD3 Female]

The shift to increasingly digital healthcare services, whilst welcomed by some, raised concerns about the impact of digital inequalities as a result of low levels of digital literacy and/or access to hardware and/or data:

…you can see a lot of people who … they cannot do things online, because of their long‐term medical conditions. They cannot do things on, even the mobile phones. The only thing they can possibly do is ring and then you cannot get to your GP. This happened to me. [FGD2 Male]

CO stakeholders highlighted the impact on health of low digital literacy and lack of access to equipment and data specifically within African diaspora communities:

…the limitations they [members] have through accessing say digital health … I don't think many Africans are aware or even if they are aware, sometimes they don't have the opportunity or chance or perhaps even the equipment to access that digital health, because perhaps if they use this [digital health platforms] … [it may be] a little more complicated for them … even understanding the language which is used. [SSI participant 2]

An important mitigating factor was support, usually from within third sector, to access services online:

… there's some charities who could support with laptops, mobile phones, so you can access some information about your health online, you can order your prescription online, you can do Zoom meetings yeah. [FGD2 Male]

### 3.4 Systemic level determinants

#### 3.4.1 Systemic barriers related to race/ethnicity

A few FGD participants expressed mistrust of biomedicine as a result of historical medical racism, which made them reluctant to seek medical care:

A couple more [of] these other factors which I believe could strongly have affected my personal uptake or the care I receive. Historically we, coming from Africa … I think there have been experiments done in the way towards Black people. You hear about the cases of syphilis and so forth … [it's a] very hard call for someone to come up front and be willing to start a medication of what they are not sure of putting in their bodies. So historical experiences are one of the factors … how do you expect me as an African in this country to buy into the idea that really what they are doing with my body could be for my good? [FGD4 Male]

This situation was further exacerbated by experiences of culturally or racially based stigma and discrimination within healthcare settings:

… when they [CO members] access generic [non‐HIV‐related] services they feel they are judged [by NHS staff], just because of the misunderstandings of their beliefs [e.g., prescribed treatment] and their norms and so on … really, we don't have services that are particularly tailored to particular groups of people, e.g., Africans. [SSI participant 3]

## 4 DISCUSSION

To the best of our knowledge, this is the first qualitative study in the UK that explores LTCs in people of Black ethnicities in the United Kingdom, and the role of SDoH on risk, outcomes, and management (including self‐management) of LTCs.

FGD and SSI participants reported that LTCs in addition to HIV were common in people living with HIV of Black ethnicities. Their accounts reveal how SDoH, including race and immigration status, result in poor mental health and constrain people's ability to adapt their lifestyles to prevent poor health outcomes. The intrinsic link between race, physical health and mental health is captured by the concept of ‘weathering’. ‘Weathering’ accelerates ageing due to early health deterioration as a result of material hardship and racism in marginalised populations.^21^ Our data show how weathering may be embodied by people of Black ethnicities living with HIV in London, for example through accumulated levels of physiological stress when dealing with racism and material deprivation.

Our data reveal that despite the burden of LTCs in this population, there remains limited awareness of the risk and potential preventative strategies. FGD and SSI participants highlight how a variety of SDoH (including economic hardship, low educational status, insecure immigration status, racially minoritised status, cultural difference, and crucially HIV‐related stigma) are perceived to increase not only the risk of LTCs (often as a result of impact on lifestyle), but also LTC outcomes, self‐management and engagement with healthcare. As a result, the CO became a trusted, valued and safe space within which people could receive stigma‐free, culturally appropriate support.

General Practitioners and specialist HIV services are aware of the complex intersection between health, social and economic marginalisation. Many HIV services screen for social vulnerability, as recommended by national HIV monitoring guidelines.^22^ However, lack of resources in an increasing cash constrained health service often prevents healthcare staff from providing holistic and adequate ongoing support.^23^ What is notably absent in these data are accounts of shared and coordinated care across primary and secondary care, which improve management of LTCs.

FGD participants reported that they felt under‐confident in navigating the healthcare system and in challenging stigma and discrimination by healthcare providers, leaving them feeling frustrated and uncared for. In addition, advice from healthcare providers were sometimes difficult to implement as a result of different cultural practices, health beliefs and/or socioeconomic deprivation. A recent *Lancet* series on race and health^24^ highlights the importance of addressing inequities to improve population health outcomes, acknowledging the long‐standing structural and institutional realities of racism, xenophobia, and discrimination within health systems. The authors propose multi‐level interventions to tackle the negative health effects of these social dimensions, across individual, community, institutional, and health system tiers. Data from this study point to a need for healthcare providers to situate patient care in the context of people's lived experience. Of particular importance is the need for staff to improve their cultural competence and receive training in antiracist practices.

Stigma is a globally recognised barrier to health seeking behaviour, engagement in care and adherence to treatment^25^, ^26^ across a range of LTCs,^27^ including HIV infection,^28^ diabetes,^29^ epilepsy.^30^ Stigma continues to have a devastating impact on people living with HIV, which one stakeholder described as ‘killing more people than HIV itself’. The study data reveal how felt and enacted stigma and discrimination (as a result of HIV and/or race) impact consultations and function as barriers to care seeking and engagement in care for LTCs.

**Figure 1:**
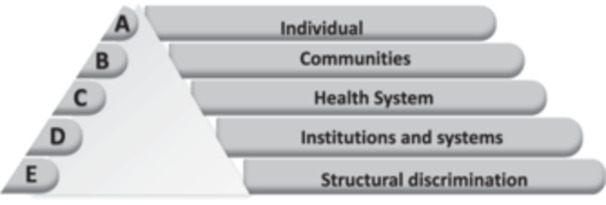
Multilevel intervention matrix for adaptation to improve the care for people with HIV and LTCs. Tier A: provision of regular access to integrated primary and secondary care, access to employment opportunities. Tier B: addressing HIV‐related stigma and discrimination through bespoke interventions within cultural context in close collaboration with people diagnosed with HIV, provision of education about LTCs and how best to navigate the NHS to community organisation in close collaboration with members. Tier C: training of NHS staff in cultural competencies, for interview panels mitigation racial bias, clinical practice, and research staff recruitment, addressing material hardship through social care. Tier D: provision of assistance to rapid benefits, immigration and housing support. Tier E: implementation of minimum wage laws, affirmative action, strengthening human rights interventions.

Based on findings from this study, we have adapted the multi‐level intervention matrix (see Figure 1) outlined by the recent Lancet series on race and health,^24^ to offer potential targets for interventions to improve LTC care amongst people living with HIV.

## 5 STRENGTHS AND LIMITATIONS OF THE STUDY

A strength of this study is the inclusion of people living with HIV as participants as well as a CO with many years of experience supporting people living with HIV from Black communities in London. The degree of overlap between these accounts from members and stakeholders was particularly revealing and provides further confidence in the validity of the findings. Moreover, the involvement of CO members and staff in the study design, data gathering, analysis and interpretation increased the relevance and rigour of the study.

FGDs and SSIs were conducted with one CO, due to time, resource and funding constraints, and COVID‐19 restrictions. It is important to note that none of the FGD participants worked full‐time, most were of East or Southern African heritage, and no one was younger than 35 years old. Therefore, the findings cannot be generalised to younger people. As this study was conducted in London the findings may not be applicable to other settings.

## 6 CONCLUSIONS AND RECOMMENDATIONS

This study will allow healthcare providers, commissioners, and policy makers to better understand the complexities of living with HIV and preventing or managing other LTCs, when also being of racially minoritised status.

Multilevel interventions are required if care is to be improved for those living with HIV and other LTCs, including to address the negative impacts of SDoH. However, this is challenging in the context of reductions in social care and health budgets, an increasingly hostile environment for migrants and reduced funding for vital COs (leading to widespread closures of local charities that provide essential services to the most marginalised groups in society).

This study has highlighted the need to co‐produce tailored and accessible educational interventions for LTCs for and with people of Black African and Black Caribbean ethnicities living with HIV in the United Kingdom. Initial findings from this study have been presented to the collaborating CO and will inform future development of resources for staff and service users. This includes specific support in shared decision‐making and challenging healthcare providers. It is vital that COs receive funding so they can continue to provide services and support that empower racially minoritised people living with HIV to achieve optimal health and wellbeing.

## AUTHOR CONTRIBUTIONS

**Vlad Kolodin**: Methodology; formal analysis; data curation; writing—review and editing; conceptualisation; validation; software. **Birgit Barbini**: Methodology; writing—review and editing; formal analysis; software. **Denis Onyango**: Methodology; writing—review and editing; validation; resources. **Rachel Musomba**: Methodology; validation; writing—review and editing. **Jia Liu**: Validation; writing—review and editing; methodology. **Rachel K. Y. Hung**: Methodology; writing—review and editing; validation. **Elena Nikiphorou**: Writing—review and editing; methodology. **Lucy Campbell**: Project administration; writing—review and editing. **Frank A. Post**: Funding acquisition; writing—review and editing. **Shema Tariq**: Writing—original draft; writing—review and editing; methodology; conceptualisation; formal analysis; validation. **Heidi Lempp**: Conceptualisation; funding acquisition; methodology; validation; writing—original draft; writing—review and editing; formal analysis; supervision; data curation.

## CONFLICT OF INTEREST STATEMENT

The authors declare no conflict of interest.

## ETHICS STATEMENT

Ethics approval was received from the Kings College London Research Ethics Committee (Reference HR/DP‐21/22‐26315) 10 December 2021. Written information about the study was provided to all participants in advance before the interviews and focus groups. Each meeting started with the researcher providing a brief overview of the research, and participants were encouraged to ask questions about the study. All participants provided written informed consent.

## Data Availability

The primary data generated that support the findings of this study are available from the senior author (Heidi Lempp) upon reasonable request.

## APPENDIX A INTERVIEW AND FOCUS GROUP SCHEDULES

### A.1

1. Interview schedule

(1) Could you please tell me about your role and job?
  – How long have you been working in this organisation/area?
  – What experience do you have working with people of African Ancestry living with HIV?
  – What experience do you have working with people of African Ancestry living with LTC?
(2) What kind of long‐term health conditions have you seen amongst people of African ancestry living with HIV in your organisation?
  – In your experience, which are the most common ones?
  – In your experience, which are the ones most difficult to live with, and the most difficult to manage by the members?
(3) In your experience, what socioeconomic circumstances may impact on the health of people of African ancestry living with HIV?
  *Probe*:
  – Age, diet, access to food, being overweight, lack of exercises?
  – Work, earnings, housing situation?
  – Relationships with family, friends, partner?
  – Access to health and social care services?
  – Stigma/discrimination, what kind of discrimination race, lower socioeconomical level
  – are these HIV related?
(4) In your view, what contributes to the development of long‐term conditions (e.g. diabetes, kidney disease, heart disease) in people of African ancestry?
  *Probe*:
  – access to health services?
  – lack of knowledge how best to manage additional long‐term conditions?
  – low income, lack of adequate food, housing?
(5) What kind of support do you think people of African ancestry need to prevent the development of LTC?
  *Probe*:
  – Strengthening of available informal community resources?
  – Additional education about LTCs?
  – Access to health and social care?
  – Financial support?
(6) In your view, what kind of existing services or resources work well for people of African ancestry living with LTC, and what needs to be improved?
  *Probe*:
  – Specialist clinics
  – community services
  – primary care access
  – information about health and social care infrastructure and access
  – educational programmes
  – informal community groups
  – access to social media
(7) In your view, what kind of services or resources that are currently not available (or being accessed) might work well for people of African ancestry living with LTC?
(8) Do you wish to provide me with any further information that I did not include in this interview schedule, and you think is important?

2. Focus group schedule

Long‐term conditions in people of African ancestry

(1) What comes to your mind when you think about long‐term conditions?
  As a group or split into two smaller groups (depends on, whether online or face to face, aided with ‘post‐its’ notes for participants to write their thoughts/knowledge)
(2) What does the term long‐term condition mean to you?
  Please give some examples.
  How do these long‐term condition/s affect your live?
  (co‐facilitator to make notes of the answers)
(3) What things do you think lead to the development of long‐term health problems?
  (Consider small group work again, subject whether rapport has been well established)
  *Probe*: age, diet, being overweight, lack of exercise, mental health, social circumstances, stigma.
  (co‐facilitator to make notes of the answers)
(4) In what ways, if any do you think HIV affects the development and/or living with a long‐term condition?
(5) What does mental health mean to you and in what ways does it affect your physical health?
  *Probe*: low self‐motivation, taking care of yourself, lack of concentration.
(6) Living with long‐term conditions
  Can you please share with us your experiences of living with long‐term conditions (diabetes, kidney disease, heart disease, stroke)?
  *Probes*:
  – How do these affect your daily lives? Which aspects of these LTC are the most challenging for you?
  – Taking medicines, regular clinic visits, seeing the GP
  – What specific challenges in your life affect your LTC:
  – low income, adequate food, work, community support, housing, mental health.
(7) In what ways does living with HIV and another long‐term condition affect your life?
  *Probes*:
  – Is it different compared with people without HIV? Or people without LTC?
  – How do you think your illnesses impact on your relationships with your family, partners?
  – What kind of positive/negative aspects you can think about? (probe: on HIV and LTC)

− Can you provide examples of being stigmatised by others because of being HIV‐positive or because of long‐term conditions? (probe: family, neighbours, friends)
  – How do you keep yourself well?

(8) *Probes*:
  – What aspects of remaining well are working for you and which ones not so well?
  – (Information, healthy lifestyle, diet, taking medicine, stick to medication regime)
  – How do you ensure that self‐management of your LTC can be integrated in your daily routines?
  – You said that X can help for Y, please tell me more about why that is the case.
(9) In your experience what aspects get in your way of staying well?
  *Probes*:
  − Is there anything preventing you from caring for yourself?
  − Are there reasons why you do not want to receive prescribed treatment or follow your doctor/nurse recommendations?
  − Who or where do go when you need financial support, housing advice, mental health support? (e.g. characteristics of person/group).
(10) Engagement with other sources of support
  Where do you go for help when you become unwell?
  *Probes*:
  – social or peer support, online information
  – your experiences of facilitation and barriers
(11) Where do you usually go for care when you need help for your long‐term conditions?
  *Probe*:
  – What barriers do you come across/do you need to overcome to access treatment?

For example, access to online services due to Covid.

Separate male and female groups

(12) What does it mean to be a man/woman living with HIV and long‐term health conditions?
(13) My final question: is there anything you would like to add that I have not asked you and is important to you?
